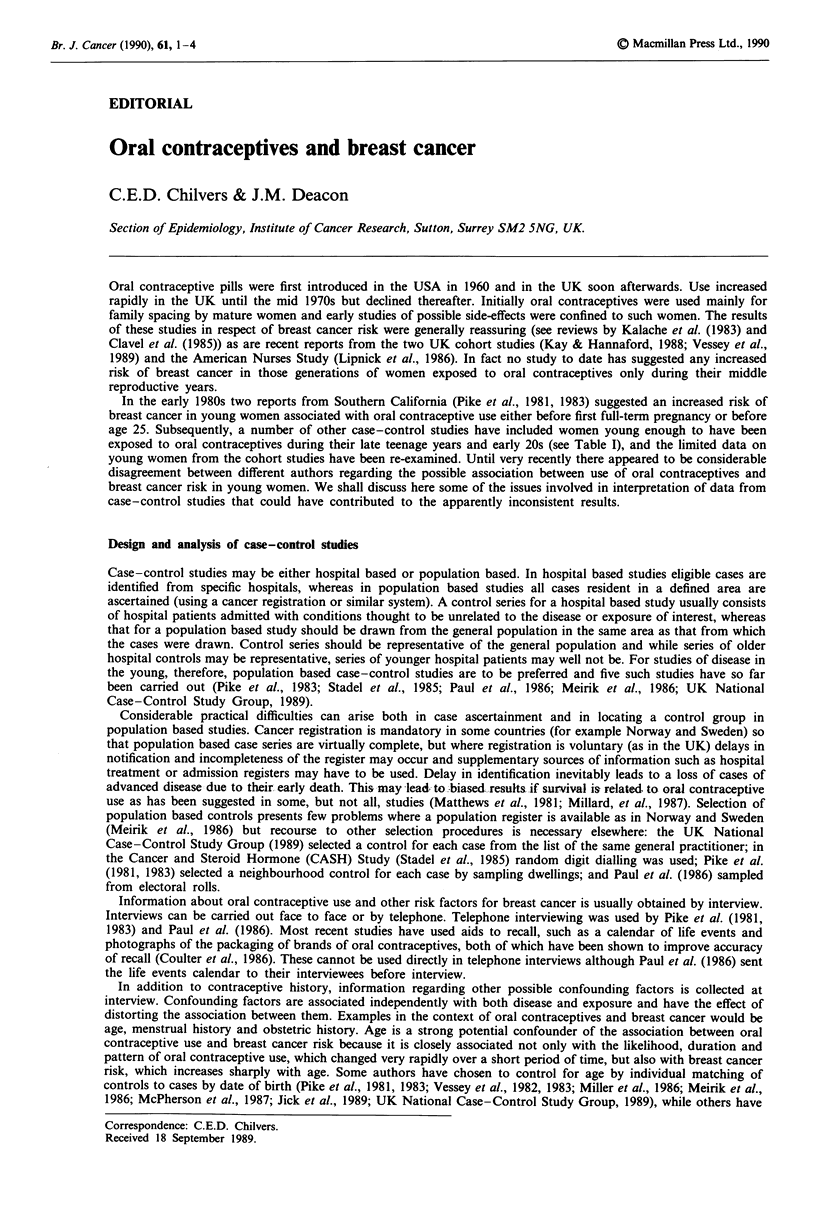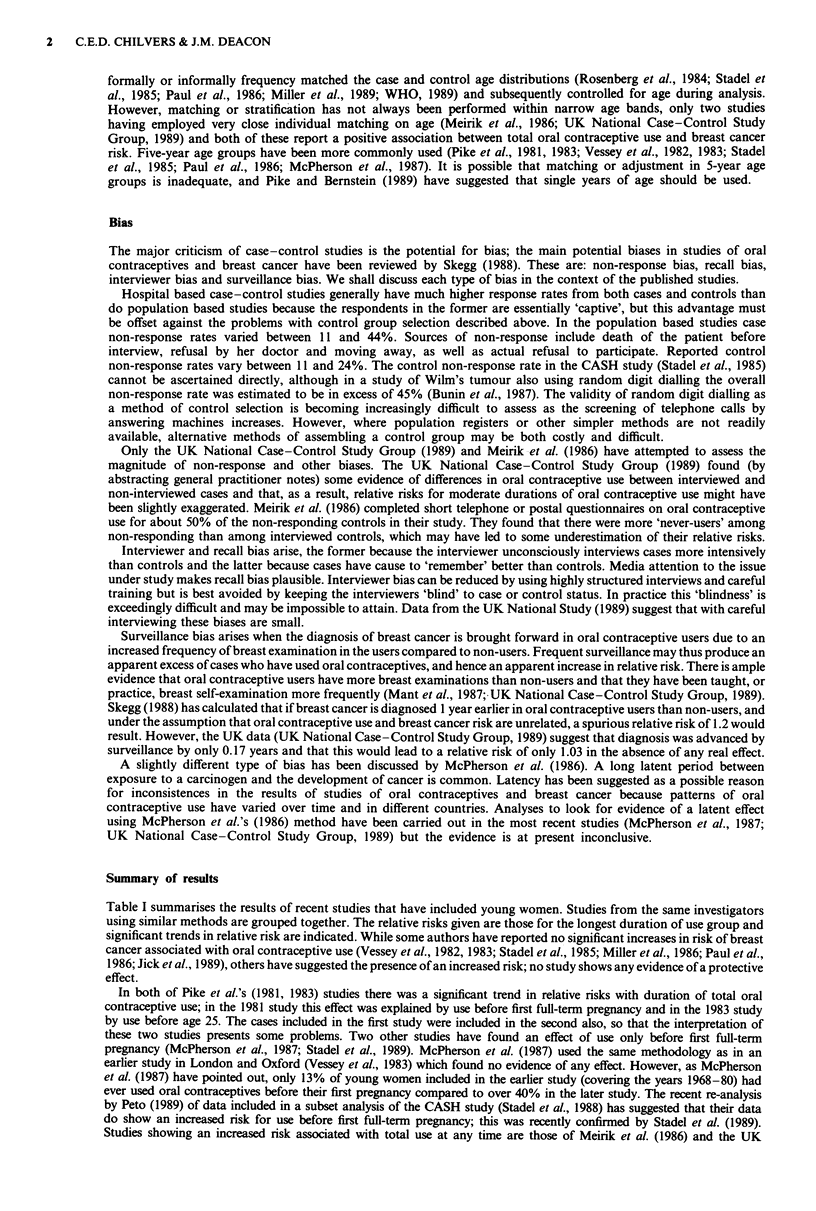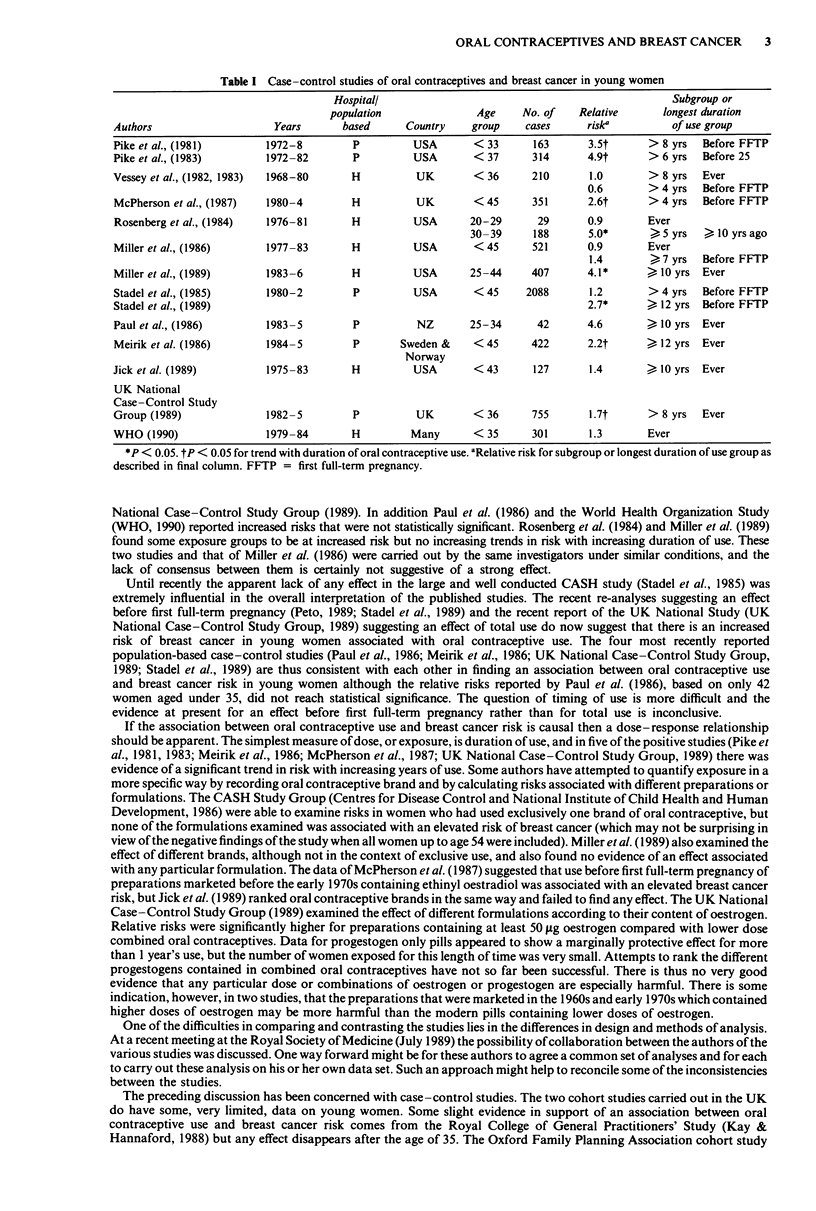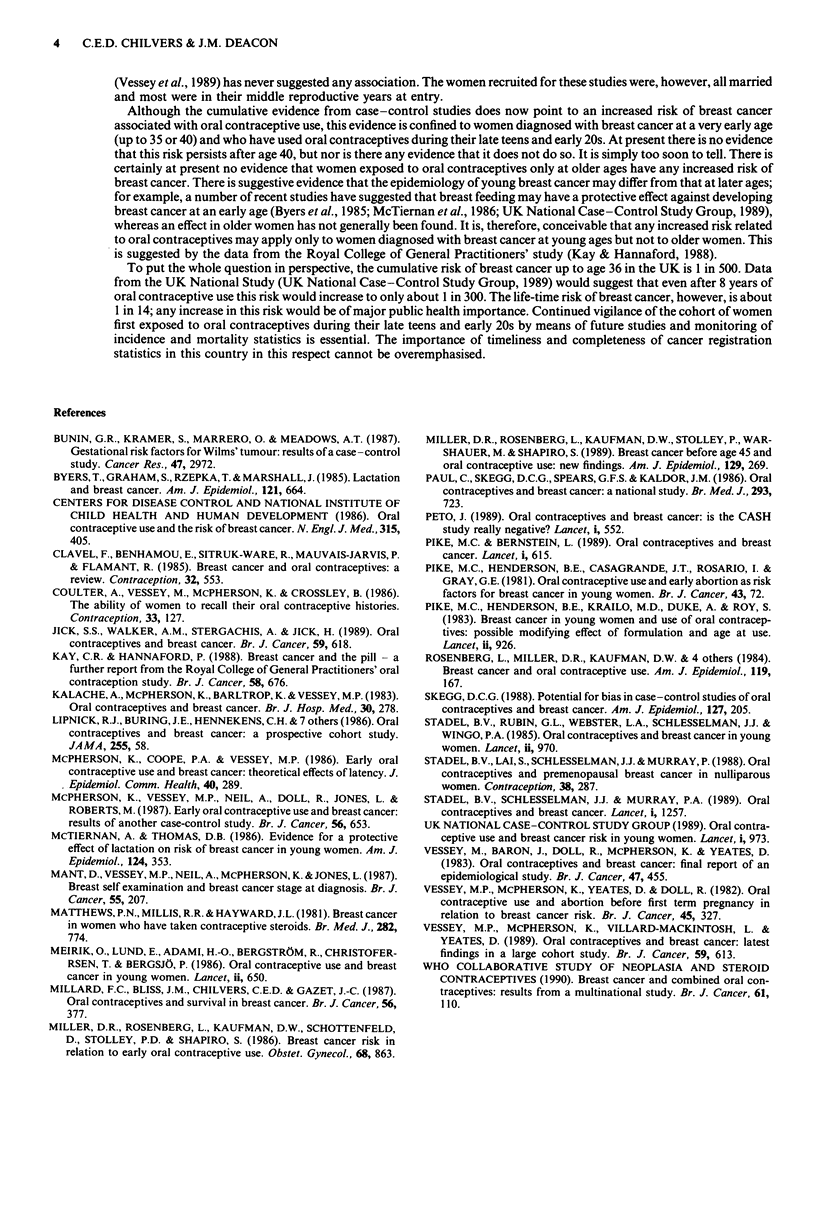# Oral contraceptives and breast cancer.

**DOI:** 10.1038/bjc.1990.1

**Published:** 1990-01

**Authors:** C. E. Chilvers, J. M. Deacon

**Affiliations:** Section of Epidemiology, Institute of Cancer Research, Sutton, Surrey, UK.


EDITORIAL

### Oral contraceptives and breast cancer

C.E.D. Chilvers & J.M. Deacon

*Section of Epidemiology, Institute of Cancer Research, Sutton, Surrey SM2 5NG, UK.*

Oral contraceptive pills were first introduced in the USA in 1960 and in the UK soon afterwards. Use increased rapidly in the UK until the mid 1970s but declined thereafter. Initially oral contraceptives were used mainly for family spacing by mature women and early studies of possible side-effects were confined to such women. The results of these studies in respect of breast cancer risk were generally reassuring (see reviews by Kalache *et al.* (1983) and Clavel *et al.* (1985)) as are recent reports from the two UK cohort studies (Kay & Hannaford, 1988; Vessey *et al.*, 1989) and the American Nurses Study (Lipnick *et al.*, 1986). In fact no study to date has suggested any increased risk of breast cancer in those generations of women exposed to oral contraceptives only during their middle reproductive years.

In the early 1980s two reports from Southern California (Pike *et al.*, 1981, 1983) suggested an increased risk of breast cancer in young women associated with oral contraceptive use either before first full-term pregnancy or before age 25. Subsequently, a number of other case-control studies have included women young enough to have been exposed to oral contraceptives during their late teenage years and early 20s (see Table I), and the limited data on young women from the cohort studies have been re-examined. Until very recently there appeared to be considerable disagreement between different authors regarding the possible association between use of oral contraceptives and breast cancer risk in young women. We shall discuss here some of the issues involved in interpretation of data from case-control studies that could have contributed to the apparently inconsistent results.

### Design and analysis of case-control studies

Case-control studies may be either hospital based or population based. In hospital based studies eligible cases are identified from specific hospitals, whereas in population based studies all cases resident in a defined area are ascertained (using a cancer registration or similar system). A control series for a hospital based study usually consists of hospital patients admitted with conditions thought to be unrelated to the disease or exposure of interest, whereas that for a population based study should be drawn from the general population in the same area as that from which the cases were drawn. Control series should be representative of the general population and while series of older hospital controls may be representative, series of younger hospital patients may well not be. For studies of disease in the young, therefore, population based case-control studies are to be preferred and five such studies have so far been carried out (Pike *et al.*, 1983; Stadel *et al.*, 1985; Paul *et al.*, 1986; Meirik *et al.*, 1986; UK National Case-Control Study Group, 1989).

Considerable practical difficulties can arise both in case ascertainment and in locating a control group in population based studies. Cancer registration is mandatory in some countries (for example Norway and Sweden) so that population based case series are virtually complete, but where registration is voluntary (as in the UK) delays in notification and incompleteness of the register may occur and supplementary sources of information such as hospital treatment or admission registers may have to be used. Delay in identification inevitably leads to a loss of cases of advanced disease due to their early death. This may lead to biased results if survival is related to oral contraceptive use as has been suggested in some, but not all, studies (Matthews *et al.*, 1981; Millard, *et al.*, 1987). Selection of population based controls presents few problems where a population register is available as in Norway and Sweden (Meirik *et al.*, 1986) but recourse to other selection procedures is necessary elsewhere: the UK National Case-Control Study Group (1989) selected a control for each case from the list of the same general practitioner; in the Cancer and Steroid Hormone (CASH) Study (Stadel *et al.*, 1985) random digit dialling was used; Pike *et al.* (1981, 1983) selected a neighbourhood control for each case by sampling dwellings; and Paul *et al.* (1986) sampled from electoral rolls.

Information about oral contraceptive use and other risk factors for breast cancer is usually obtained by interview. Interviews can be carried out face to face or by telephone. Telephone interviewing was used by Pike *et al.* (1981, 1983) and Paul *et al.* (1986). Most recent studies have used aids to recall, such as a calendar of life events and photographs of the packaging of brands of oral contraceptives, both of which have been shown to improve accuracy of recall (Coulter *et al.*, 1986). These cannot be used directly in telephone interviews although Paul *et al.* (1986) sent the life events calendar to their interviewees before interview.

In addition to contraceptive history, information regarding other possible confounding factors is collected at interview. Confounding factors are associated independently with both disease and exposure and have the effect of distorting the association between them. Examples in the context of oral contraceptives and breast cancer would be age, menstrual history and obstetric history. Age is a strong potential confounder of the association between oral contraceptive use and breast cancer risk because it is closely associated not only with the likelihood, duration and pattern of oral contraceptive use, which changed very rapidly over a short period of time, but also with breast cancer risk, which increases sharply with age. Some authors have chosen to control for age by individual matching of controls to cases by date of birth (Pike *et al.*, 1981, 1983; Vessey *et al.*, 1982, 1983; Miller *et al.*, 1986; Meirik *et al.*, 1986; McPherson *et al.*, 1987; Jick *et al.*, 1989; UK National Case-Control Study Group, 1989), while others have

Correspondence: C.E.D. Chilvers.
Received 18 September 1989.

formally or informally frequency matched the case and control age distributions (Rosenberg *et al.*, 1984; Stadel *et al.*, 1985; Paul *et al.*, 1986; Miller *et al.*, 1989; WHO, 1989) and subsequently controlled for age during analysis. However, matching or stratification has not always been performed within narrow age bands, only two studies having employed very close individual matching on age (Meirik *et al.*, 1986; UK National Case-Control Study Group, 1989) and both of these report a positive association between total oral contraceptive use and breast cancer risk. Five-year age groups have been more commonly used (Pike *et al.*, 1981, 1983; Vessey *et al.*, 1982, 1983; Stadel *et al.*, 1985; Paul *et al.*, 1986; McPherson *et al.*, 1987). It is possible that matching or adjustment in 5-year age groups is inadequate, and Pike and Bernstein (1989) have suggested that single years of age should be used.

**Bias**

The major criticism of case-control studies is the potential for bias; the main potential biases in studies of oral contraceptives and breast cancer have been reviewed by Skegg (1988). These are: non-response bias, recall bias, interviewer bias and surveillance bias. We shall discuss each type of bias in the context of the published studies.

Hospital based case-control studies generally have much higher response rates from both cases and controls than do population based studies because the respondents in the former are essentially 'captive', but this advantage must be offset against the problems with control group selection described above. In the population based studies case non-response rates varied between 11 and 44%. Sources of non-response include death of the patient before interview, refusal by her doctor and moving away, as well as actual refusal to participate. Reported control non-response rates vary between 11 and 24%. The control non-response rate in the CASH study (Stadel *et al.*, 1985) cannot be ascertained directly, although in a study of Wilm's tumour also using random digit dialling the overall non-response rate was estimated to be in excess of 45% (Bunin *et al.*, 1987). The validity of random digit dialling as a method of control selection is becoming increasingly difficult to assess as the screening of telephone calls by answering machines increases. However, where population registers or other simpler methods are not readily available, alternative methods of assembling a control group may be both costly and difficult.

Only the UK National Case-Control Study Group (1989) and Meirik *et al.* (1986) have attempted to assess the magnitude of non-response and other biases. The UK National Case-Control Study Group (1989) found (by abstracting general practitioner notes) some evidence of differences in oral contraceptive use between interviewed and non-interviewed cases and that, as a result, relative risks for moderate durations of oral contraceptive use might have been slightly exaggerated. Meirik *et al.* (1986) completed short telephone or postal questionnaires on oral contraceptive use for about 50% of the non-responding controls in their study. They found that there were more 'never-users' among non-responding than among interviewed controls, which may have led to some underestimation of their relative risks.

Interviewer and recall bias arise, the former because the interviewer unconsciously interviews cases more intensively than controls and the latter because cases have cause to 'remember' better than controls. Media attention to the issue under study makes recall bias plausible. Interviewer bias can be reduced by using highly structured interviews and careful training but is best avoided by keeping the interviewers 'blind' to case or control status. In practice this 'blindness' is exceedingly difficult and may be impossible to attain. Data from the UK National Study (1989) suggest that with careful interviewing these biases are small.

Surveillance bias arises when the diagnosis of breast cancer is brought forward in oral contraceptive users due to an increased frequency of breast examination in the users compared to non-users. Frequent surveillance may thus produce an apparent excess of cases who have used oral contraceptives, and hence an apparent increase in relative risk. There is ample evidence that oral contraceptive users have more breast examinations than non-users and that they have been taught, or practice, breast self-examination more frequently (Mant *et al.*, 1987; UK National Case-Control Study Group, 1989). Skegg (1988) has calculated that if breast cancer is diagnosed 1 year earlier in oral contraceptive users than non-users, and under the assumption that oral contraceptive use and breast cancer risk are unrelated, a spurious relative risk of 1.2 would result. However, the UK data (UK National Case-Control Study Group, 1989) suggest that diagnosis was advanced by surveillance by only 0.17 years and that this would lead to a relative risk of only 1.03 in the absence of any real effect.

A slightly different type of bias has been discussed by McPherson *et al.* (1986). A long latent period between exposure to a carcinogen and the development of cancer is common. Latency has been suggested as a possible reason for inconsistences in the results of studies of oral contraceptives and breast cancer because patterns of oral contraceptive use have varied over time and in different countries. Analyses to look for evidence of a latent effect using McPherson *et al.*'s (1986) method have been carried out in the most recent studies (McPherson *et al.*, 1987; UK National Case-Control Study Group, 1989) but the evidence is at present inconclusive.

**Summary of results**

Table I summarises the results of recent studies that have included young women. Studies from the same investigators using similar methods are grouped together. The relative risks given are those for the longest duration of use group and significant trends in relative risk are indicated. While some authors have reported no significant increases in risk of breast cancer associated with oral contraceptive use (Vessey *et al.*, 1982, 1983; Stadel *et al.*, 1985; Miller *et al.*, 1986; Paul *et al.*, 1986; Jick *et al.*, 1989), others have suggested the presence of an increased risk; no study shows any evidence of a protective effect.

In both of Pike *et al.*'s (1981, 1983) studies there was a significant trend in relative risks with duration of total oral contraceptive use; in the 1981 study this effect was explained by use before first full-term pregnancy and in the 1983 study by use before age 25. The cases included in the first study were included in the second also, so that the interpretation of these two studies presents some problems. Two other studies have found an effect of use only before first full-term pregnancy (McPherson *et al.*, 1987; Stadel *et al.*, 1989). McPherson *et al.* (1987) used the same methodology as in an earlier study in London and Oxford (Vessey *et al.*, 1983) which found no evidence of any effect. However, as McPherson *et al.* (1987) have pointed out, only 13% of young women included in the earlier study (covering the years 1968-80) had ever used oral contraceptives before their first pregnancy compared to over 40% in the later study. The recent re-analysis by Peto (1989) of data included in a subset analysis of the CASH study (Stadel *et al.*, 1988) has suggested that their data do show an increased risk for use before first full-term pregnancy; this was recently confirmed by Stadel *et al.* (1989). Studies showing an increased risk associated with total use at any time are those of Meirik *et al.* (1986) and the UK

**Table I** Case–control studies of oral contraceptives and breast cancer in young women

| *Authors* | *Years* | *Hospital/ population based* | *Country* | *Age group* | *No. of cases* | *Relative risk*[a] | *Subgroup or longest duration of use group* | |
|---|---|---|---|---|---|---|---|---|
| Pike *et al.*, (1981) | 1972–8 | P | USA | < 33 | 163 | 3.5† | > 8 yrs | Before FFTP |
| Pike *et al.*, (1983) | 1972–82 | P | USA | < 37 | 314 | 4.9† | > 6 yrs | Before 25 |
| Vessey *et al.*, (1982, 1983) | 1968–80 | H | UK | < 36 | 210 | 1.0 | > 8 yrs | Ever |
| | | | | | | 0.6 | > 4 yrs | Before FFTP |
| McPherson *et al.*, (1987) | 1980–4 | H | UK | < 45 | 351 | 2.6† | > 4 yrs | Before FFTP |
| Rosenberg *et al.*, (1984) | 1976–81 | H | USA | 20–29 | 29 | 0.9 | Ever | |
| | | | | 30–39 | 188 | 5.0* | ⩾ 5 yrs | ⩾ 10 yrs ago |
| Miller *et al.*, (1986) | 1977–83 | H | USA | < 45 | 521 | 0.9 | Ever | |
| | | | | | | 1.4 | ⩾ 7 yrs | Before FFTP |
| Miller *et al.*, (1989) | 1983–6 | H | USA | 25–44 | 407 | 4.1* | ⩾ 10 yrs | Ever |
| Stadel *et al.*, (1985) | 1980–2 | P | USA | < 45 | 2088 | 1.2 | > 4 yrs | Before FFTP |
| Stadel *et al.*, (1989) | | | | | | 2.7* | ⩾ 12 yrs | Before FFTP |
| Paul *et al.*, (1986) | 1983–5 | P | NZ | 25–34 | 42 | 4.6 | ⩾ 10 yrs | Ever |
| Meirik *et al.* (1986) | 1984–5 | P | Sweden & Norway | < 45 | 422 | 2.2† | ⩾ 12 yrs | Ever |
| Jick *et al.* (1989) | 1975–83 | H | USA | < 43 | 127 | 1.4 | ⩾ 10 yrs | Ever |
| UK National Case–Control Study Group (1989) | 1982–5 | P | UK | < 36 | 755 | 1.7† | > 8 yrs | Ever |
| WHO (1990) | 1979–84 | H | Many | < 35 | 301 | 1.3 | Ever | |

*$P < 0.05$. †$P < 0.05$ for trend with duration of oral contraceptive use. [a]Relative risk for subgroup or longest duration of use group as described in final column. FFTP = first full-term pregnancy.

National Case–Control Study Group (1989). In addition Paul *et al.* (1986) and the World Health Organization Study (WHO, 1990) reported increased risks that were not statistically significant. Rosenberg *et al.* (1984) and Miller *et al.* (1989) found some exposure groups to be at increased risk but no increasing trends in risk with increasing duration of use. These two studies and that of Miller *et al.* (1986) were carried out by the same investigators under similar conditions, and the lack of consensus between them is certainly not suggestive of a strong effect.

Until recently the apparent lack of any effect in the large and well conducted CASH study (Stadel *et al.*, 1985) was extremely influential in the overall interpretation of the published studies. The recent re-analyses suggesting an effect before first full-term pregnancy (Peto, 1989; Stadel *et al.*, 1989) and the recent report of the UK National Study (UK National Case–Control Study Group, 1989) suggesting an effect of total use do now suggest that there is an increased risk of breast cancer in young women associated with oral contraceptive use. The four most recently reported population-based case–control studies (Paul *et al.*, 1986; Meirik *et al.*, 1986; UK National Case–Control Study Group, 1989; Stadel *et al.*, 1989) are thus consistent with each other in finding an association between oral contraceptive use and breast cancer risk in young women although the relative risks reported by Paul *et al.* (1986), based on only 42 women aged under 35, did not reach statistical significance. The question of timing of use is more difficult and the evidence at present for an effect before first full-term pregnancy rather than for total use is inconclusive.

If the association between oral contraceptive use and breast cancer risk is causal then a dose–response relationship should be apparent. The simplest measure of dose, or exposure, is duration of use, and in five of the positive studies (Pike *et al.*, 1981, 1983; Meirik *et al.*, 1986; McPherson *et al.*, 1987; UK National Case–Control Study Group, 1989) there was evidence of a significant trend in risk with increasing years of use. Some authors have attempted to quantify exposure in a more specific way by recording oral contraceptive brand and by calculating risks associated with different preparations or formulations. The CASH Study Group (Centres for Disease Control and National Institute of Child Health and Human Development, 1986) were able to examine risks in women who had used exclusively one brand of oral contraceptive, but none of the formulations examined was associated with an elevated risk of breast cancer (which may not be surprising in view of the negative findings of the study when all women up to age 54 were included). Miller *et al.* (1989) also examined the effect of different brands, although not in the context of exclusive use, and also found no evidence of an effect associated with any particular formulation. The data of McPherson *et al.* (1987) suggested that use before first full-term pregnancy of preparations marketed before the early 1970s containing ethinyl oestradiol was associated with an elevated breast cancer risk, but Jick *et al.* (1989) ranked oral contraceptive brands in the same way and failed to find any effect. The UK National Case–Control Study Group (1989) examined the effect of different formulations according to their content of oestrogen. Relative risks were significantly higher for preparations containing at least 50 μg oestrogen compared with lower dose combined oral contraceptives. Data for progestogen only pills appeared to show a marginally protective effect for more than 1 year's use, but the number of women exposed for this length of time was very small. Attempts to rank the different progestogens contained in combined oral contraceptives have not so far been successful. There is thus no very good evidence that any particular dose or combinations of oestrogen or progestogen are especially harmful. There is some indication, however, in two studies, that the preparations that were marketed in the 1960s and early 1970s which contained higher doses of oestrogen may be more harmful than the modern pills containing lower doses of oestrogen.

One of the difficulties in comparing and contrasting the studies lies in the differences in design and methods of analysis. At a recent meeting at the Royal Society of Medicine (July 1989) the possibility of collaboration between the authors of the various studies was discussed. One way forward might be for these authors to agree a common set of analyses and for each to carry out these analysis on his or her own data set. Such an approach might help to reconcile some of the inconsistencies between the studies.

The preceding discussion has been concerned with case–control studies. The two cohort studies carried out in the UK do have some, very limited, data on young women. Some slight evidence in support of an association between oral contraceptive use and breast cancer risk comes from the Royal College of General Practitioners' Study (Kay & Hannaford, 1988) but any effect disappears after the age of 35. The Oxford Family Planning Association cohort study

(Vessey *et al.*, 1989) has never suggested any association. The women recruited for these studies were, however, all married and most were in their middle reproductive years at entry.

Although the cumulative evidence from case–control studies does now point to an increased risk of breast cancer associated with oral contraceptive use, this evidence is confined to women diagnosed with breast cancer at a very early age (up to 35 or 40) and who have used oral contraceptives during their late teens and early 20s. At present there is no evidence that this risk persists after age 40, but nor is there any evidence that it does not do so. It is simply too soon to tell. There is certainly at present no evidence that women exposed to oral contraceptives only at older ages have any increased risk of breast cancer. There is suggestive evidence that the epidemiology of young breast cancer may differ from that at later ages; for example, a number of recent studies have suggested that breast feeding may have a protective effect against developing breast cancer at an early age (Byers *et al.*, 1985; McTiernan *et al.*, 1986; UK National Case–Control Study Group, 1989), whereas an effect in older women has not generally been found. It is, therefore, conceivable that any increased risk related to oral contraceptives may apply only to women diagnosed with breast cancer at young ages but not to older women. This is suggested by the data from the Royal College of General Practitioners' study (Kay & Hannaford, 1988).

To put the whole question in perspective, the cumulative risk of breast cancer up to age 36 in the UK is 1 in 500. Data from the UK National Study (UK National Case–Control Study Group, 1989) would suggest that even after 8 years of oral contraceptive use this risk would increase to only about 1 in 300. The life-time risk of breast cancer, however, is about 1 in 14; any increase in this risk would be of major public health importance. Continued vigilance of the cohort of women first exposed to oral contraceptives during their late teens and early 20s by means of future studies and monitoring of incidence and mortality statistics is essential. The importance of timeliness and completeness of cancer registration statistics in this country in this respect cannot be overemphasised.

### References

BUNIN, G.R., KRAMER, S., MARRERO, O. & MEADOWS, A.T. (1987). Gestational risk factors for Wilms' tumour: results of a case–control study. *Cancer Res.*, **47**, 2972.

BYERS, T., GRAHAM, S., RZEPKA, T. & MARSHALL, J. (1985). Lactation and breast cancer. *Am. J. Epidemiol.*, **121**, 664.

CENTERS FOR DISEASE CONTROL AND NATIONAL INSTITUTE OF CHILD HEALTH AND HUMAN DEVELOPMENT (1986). Oral contraceptive use and the risk of breast cancer. *N. Engl. J. Med.*, **315**, 405.

CLAVEL, F., BENHAMOU, E., SITRUK-WARE, R., MAUVAIS-JARVIS, P. & FLAMANT, R. (1985). Breast cancer and oral contraceptives: a review. *Contraception*, **32**, 553.

COULTER, A., VESSEY, M., MCPHERSON, K. & CROSSLEY, B. (1986). The ability of women to recall their oral contraceptive histories. *Contraception*, **33**, 127.

JICK, S.S., WALKER, A.M., STERGACHIS, A. & JICK, H. (1989). Oral contraceptives and breast cancer. *Br. J. Cancer*, **59**, 618.

KAY, C.R. & HANNAFORD, P. (1988). Breast cancer and the pill – a further report from the Royal College of General Practitioners' oral contraception study. *Br. J. Cancer*, **58**, 676.

KALACHE, A., MCPHERSON, K., BARLTROP, K. & VESSEY, M.P. (1983). Oral contraceptives and breast cancer. *Br. J. Hosp. Med.*, **30**, 278.

LIPNICK, R.J., BURING, J.E., HENNEKENS, C.H. & 7 others (1986). Oral contraceptives and breast cancer: a prospective cohort study. *JAMA*, **255**, 58.

MCPHERSON, K., COOPE, P.A. & VESSEY, M.P. (1986). Early oral contraceptive use and breast cancer: theoretical effects of latency. *J. Epidemiol. Comm. Health*, **40**, 289.

MCPHERSON, K., VESSEY, M.P., NEIL, A., DOLL, R., JONES, L. & ROBERTS, M. (1987). Early oral contraceptive use and breast cancer: results of another case-control study. *Br. J. Cancer*, **56**, 653.

MCTIERNAN, A. & THOMAS, D.B. (1986). Evidence for a protective effect of lactation on risk of breast cancer in young women. *Am. J. Epidemiol.*, **124**, 353.

MANT, D., VESSEY, M.P., NEIL, A., MCPHERSON, K. & JONES, L. (1987). Breast self examination and breast cancer stage at diagnosis. *Br. J. Cancer*, **55**, 207.

MATTHEWS, P.N., MILLIS, R.R. & HAYWARD, J.L. (1981). Breast cancer in women who have taken contraceptive steroids. *Br. Med. J.*, **282**, 774.

MEIRIK, O., LUND, E., ADAMI, H.-O., BERGSTRÖM, R., CHRISTOFERRSEN, T. & BERGSJÖ, P. (1986). Oral contraceptive use and breast cancer in young women. *Lancet*, **ii**, 650.

MILLARD, F.C., BLISS, J.M., CHILVERS, C.E.D. & GAZET, J.-C. (1987). Oral contraceptives and survival in breast cancer. *Br. J. Cancer*, **56**, 377.

MILLER, D.R., ROSENBERG, L., KAUFMAN, D.W., SCHOTTENFELD, D., STOLLEY, P.D. & SHAPIRO, S. (1986). Breast cancer risk in relation to early oral contraceptive use. *Obstet. Gynecol.*, **68**, 863.

MILLER, D.R., ROSENBERG, L., KAUFMAN, D.W., STOLLEY, P., WARSHAUER, M. & SHAPIRO, S. (1989). Breast cancer before age 45 and oral contraceptive use: new findings. *Am. J. Epidemiol.*, **129**, 269.

PAUL, C., SKEGG, D.C.G., SPEARS, G.F.S. & KALDOR, J.M. (1986). Oral contraceptives and breast cancer: a national study. *Br. Med. J.*, **293**, 723.

PETO, J. (1989). Oral contraceptives and breast cancer: is the CASH study really negative? *Lancet*, **i**, 552.

PIKE, M.C. & BERNSTEIN, L. (1989). Oral contraceptives and breast cancer. *Lancet*, **i**, 615.

PIKE, M.C., HENDERSON, B.E., CASAGRANDE, J.T., ROSARIO, I. & GRAY, G.E. (1981). Oral contraceptive use and early abortion as risk factors for breast cancer in young women. *Br. J. Cancer*, **43**, 72.

PIKE, M.C., HENDERSON, B.E., KRAILO, M.D., DUKE, A. & ROY, S. (1983). Breast cancer in young women and use of oral contraceptives: possible modifying effect of formulation and age at use. *Lancet*, **ii**, 926.

ROSENBERG, L., MILLER, D.R., KAUFMAN, D.W. & 4 others (1984). Breast cancer and oral contraceptive use. *Am. J. Epidemiol.*, **119**, 167.

SKEGG, D.C.G. (1988). Potential for bias in case–control studies of oral contraceptives and breast cancer. *Am. J. Epidemiol.*, **127**, 205.

STADEL, B.V., RUBIN, G.L., WEBSTER, L.A., SCHLESSELMAN, J.J. & WINGO, P.A. (1985). Oral contraceptives and breast cancer in young women. *Lancet*, **ii**, 970.

STADEL, B.V., LAI, S., SCHLESSELMAN, J.J. & MURRAY, P. (1988). Oral contraceptives and premenopausal breast cancer in nulliparous women. *Contraception*, **38**, 287.

STADEL, B.V., SCHLESSELMAN, J.J. & MURRAY, P.A. (1989). Oral contraceptives and breast cancer. *Lancet*, **i**, 1257.

UK NATIONAL CASE–CONTROL STUDY GROUP (1989). Oral contraceptive use and breast cancer risk in young women. *Lancet*, **i**, 973.

VESSEY, M., BARON, J., DOLL, R., MCPHERSON, K. & YEATES, D. (1983). Oral contraceptives and breast cancer: final report of an epidemiological study. *Br. J. Cancer*, **47**, 455.

VESSEY, M.P., MCPHERSON, K., YEATES, D. & DOLL, R. (1982). Oral contraceptive use and abortion before first term pregnancy in relation to breast cancer risk. *Br. J. Cancer*, **45**, 327.

VESSEY, M.P., MCPHERSON, K., VILLARD-MACKINTOSH, L. & YEATES, D. (1989). Oral contraceptives and breast cancer: latest findings in a large cohort study. *Br. J. Cancer*, **59**, 613.

WHO COLLABORATIVE STUDY OF NEOPLASIA AND STEROID CONTRACEPTIVES (1990). Breast cancer and combined oral contraceptives: results from a multinational study. *Br. J. Cancer*, **61**, 110.